# Molecular signatures associated with cognitive deficits in schizophrenia: a study of biopsied olfactory neural epithelium

**DOI:** 10.1038/tp.2016.154

**Published:** 2016-10-11

**Authors:** Y Horiuchi, M A Kondo, K Okada, Y Takayanagi, T Tanaka, T Ho, M Varvaris, K Tajinda, H Hiyama, K Ni, C Colantuoni, D Schretlen, N G Cascella, J Pevsner, K Ishizuka, A Sawa

**Affiliations:** 1 Department of Psychiatry, Johns Hopkins University, Baltimore, MD, USA; 2 Department of Mental Health, Johns Hopkins University, Baltimore, MD, USA; 3 Pharmacology Research Labs, Astellas Pharma Inc., Tsukuba-shi, Ibaraki, Japan; 4 Lieber Institute for Brain Development, Baltimore, MD, USA; 5 Hugo W Moser Research Institute at Kennedy Krieger, Baltimore, MD, USA

## Abstract

Cognitive impairment is a key feature of schizophrenia (SZ) and determines functional outcome. Nonetheless, molecular signatures in neuronal tissues that associate with deficits are not well understood. We conducted nasal biopsy to obtain olfactory epithelium from patients with SZ and control subjects. The neural layers from the biopsied epithelium were enriched by laser-captured microdissection. We then performed an unbiased microarray expression study and implemented a systematic neuropsychological assessment on the same participants. The differentially regulated genes in SZ were further filtered based on correlation with neuropsychological traits. This strategy identified the *SMAD 5* gene, and real-time quantitative PCR analysis also supports downregulation of the SMAD pathway in SZ. The SMAD pathway has been important in multiple tissues, including the role for neurodevelopment and bone formation. Here the involvement of the pathway in adult brain function is suggested. This exploratory study establishes a strategy to better identify neuronal molecular signatures that are potentially associated with mental illness and cognitive deficits. We propose that the SMAD pathway may be a novel target in addressing cognitive deficit of SZ in future studies.

## Introduction

Although operational diagnostic systems such as the Diagnostic and Statistical Manual of Mental Disorders (DSM) may increase the reliability of diagnoses,^1^ they do little to aid our understanding of the biological basis of psychiatric conditions. For example, cognitive impairment is a key feature of schizophrenia (SZ) and a predictor of functional outcome in patients:^2, 3, 4^ nonetheless, molecular cascades that specifically underlie cognitive deficit in SZ are not yet well understood. It is likely that the current DSM-based classification of SZ alone is insufficient for providing an accurate framework for studying molecular signatures underlying cognitive impairment.

A perpetual challenge to uncovering molecular mechanisms in psychiatric disorders is the difficulty of obtaining central nervous system tissues and cells from live patients that are likely to underlie clinical features.^5, 6, 7^ Post-mortem brains are valuable resources for studying molecular signatures; however, these do not reliably provide molecular information associated with the onset or course of cognitive impairment in living subjects. There is a critical need for reliable and representative biological samples that can be collected longitudinally. Blood is frequently used because of its ease of repeated collection; however, we have previously shown that the gene expression profile of lymphocytes and lymphoblasts (LBs) derived from peripheral blood is dissimilar to that of fetal and adult brain.^8^ Several groups have proposed that the olfactory epithelium (OE), which contains olfactory receptor neurons, may be a good surrogate to address this question.^8, 9, 10, 11^ In particular, we have reported a method to enrich neuronal cells from the biopsied epithelium using laser-captured microdissection (LCM) to study molecular signatures relevant to neurons.^12, 13^

Deficits in olfaction are known in several neuropsychiatric conditions, including SZ, Parkinson's disease and Alzheimer's disease, which likely associate with cellular or molecular dysregulation in the OE.^9, 11, 14^ In SZ, olfactory dysfunction is associated with negative and cognitive symptoms.^15, 16, 17^ Furthermore, the OE contains cells of neuronal lineage at multiple stages of maturity.^18, 19^ This provides a unique opportunity to investigate aspects of neurodevelopment, which may have been derailed early in life in SZ.

In the present study, we aimed to establish a strategy to uncover molecular signatures that reflect changes in neuronal cells and are associated with cognitive impairment in SZ. This exploratory study addressed the question using a unique, multilayered approach to discover molecular candidates, taking correlation with behavioral dimensions into consideration. We examined molecular expression profiles in the olfactory neuronal layers in parallel with systematic neuropsychological assessments on the same participants. We used correlation between differentially expressed genes and neuropsychological traits as an additional filter following correction for multiple testing. On the basis of this exploratory strategy, we propose that the SMAD pathway may be an interesting target in studying cognitive deficits related to SZ.

## Materials and methods

### Subject recruitment and clinical assessment

Patients with chronic SZ were recruited from outpatient clinics in the Johns Hopkins Schizophrenia Center. Diagnosis was determined according to the DSM-IV.^20^ Normal control subjects were recruited from the general population through flyers posted at Johns Hopkins Medicine and an *ad hoc* advertisement in a local magazine. Participants were matched for age, sex, race, education and smoking status. All subjects were administered the Structured Clinical Interview for DSM-IV Axis I Disorders-Clinician Version,^21^ and SZ patients were assessed with the Scales for the Assessment of Positive and Negative Symptoms.^22^ Subjects were excluded from the study if they had a history of traumatic brain injury with loss of consciousness for >1 h, a history of drug abuse within 6 months of the study, a history of drug dependence within 12 months of the study or a history of untreated major medical illnesses. The study was conducted under approval of the Johns Hopkins Institutional Review Board (Protocol #NA_00037204: AS is the PI of this protocol), and all the subjects gave written consent for their participation.

### Nasal biopsy and LCM of OE from subjects

OE tissues were obtained with nasal biopsy under local anesthesia to the nasal cavity. The biopsy procedure was performed under endoscopic control and used either a small curette or biting forceps for tissue removal. To avoid trauma to the cribriform plate, the biopsies were usually taken from the upper nasal septum. The tissue was removed from either the front or the back of the olfactory cleft, or both. Four to six pieces of tissue (5 mm^3^) were removed from each nostril. The neuronal OE layers were enriched and purified using LCM. Detailed methods for this procedure have been reported previously by our group^12, 13^ and can be viewed at the following URL: http://www.jove.com/video/51853/. As described previously, successful enrichment of neuronal cells using this method is evidenced by substantially higher expression of olfactory marker protein, a specific olfactory receptor neuronal marker, and beta tubulin III in the microdissected tissue relative to that of the whole OE sample.^13^

### Neuropsychological assessment

Neuropsychological assessments were conducted for 16 SZ patients and 15 control subjects (demographic data are shown in Table 1) according to a methodology published by our group.^16, 23, 24^ Participants completed tests from the Calibrated Neuropsychological Normative System and the Hopkins Adult Reading Test (HART) to estimate premorbid IQ based on the HART. The six-factor domain includes the following: (i) (psychomotor) processing speed, (Trail Making Test–part A, Trail Making Test–part B and Grooved Pegboard Test); (ii) attention/working memory (Brief Test of Attention–Letter, Brief Test of Attention–Number and Conners' Continuous Performance Test hit reaction time s.e.); (iii) verbal learning/memory, (Hopkins Verbal Learning Test-Revised–Learning and Hopkins Verbal Learning Test–Delayed Recall); (iv) visual learning/memory (Brief Visuospatial Memory Test-Revised–Learning and Brief Visuospatial Memory Test-Revised–Delayed Recall); (v) ideational fluency (letter, category and design tests); and (vi) executive functioning (Modified Wisconsin Card Sorting Test–category sorts and Modified Wisconsin Card Sorting Test–errors).^24^ The neuropsychological test scores in each domain have acceptable internal homogeneity, as shown in our previous study.^23^ The six factors capture the fundamental domains of cognitive functioning typically disrupted in SZ and the composite score is defined as the mean of the six-factor scores.^24, 25^

### LBs from human subjects

Peripheral blood samples were collected from all subjects who underwent nasal biopsy and neuropsychological assessments (except for one SZ patient from whom we had OE but could not obtain blood). Lymphocytes were obtained from the peripheral blood, followed by incubation at 37 °C for 20–25 days to generate LBs according to a published protocol.^26^ As the LBs were derived from subjects who also underwent nasal biopsy, all of the LB samples were 'paired' with their respective OE tissue. Therefore, they are hereafter referred to as the 'paired' LBs (15 patients with SZ and 15 control subjects).

In addition, LBs were generated from peripheral blood of an independent cohort consisting of 16 patients with SZ and 18 control subjects using the method described above. These LBs are hereafter referred to as the 'non-paired' LBs to distinguish them from the 'paired' LBs (see Table 1).

### Microarray analysis

We used a published protocol from our group.^14^ Briefly, total RNA was isolated from the neural layers of OE or paired/non-paired LBs from patients with SZ and control subjects using the RNeasy Mini Kit (Qiagen) according to manufacturer's instructions. The RNA was of high quality, with an RNA integrity number score of 10, which was assessed using a Bioanalyzer RNA 6000 Nano Chip (Agilent Technologies). Fragmented biotin-labeled cRNAs were hybridized to Affymetrix U133 Plus2.0 according to the manufacturer's protocol. Data analysis was performed using the Partek Genomics Suite software (version 6.5, Partek) and R (http://www.r-project.org/, version 3.1.1) with Bioconductor packages (http://www.bioconductor.org/). Raw intensities were normalized using the GC-Robust Multi-array Average. For differential gene expression analysis, one-way analysis of variance (ANOVA) was used to test the mean differences between two groups. The ANOVA *P*-values were adjusted using the Benjamini–Hochberg procedure to control the false discovery rate (FDR).^27^ The raw data (CEL files) are deposited in the Gene Expression Omnibus archive at the National Center for Biotechnology Information (GSE73129: http://www.ncbi.nlm.nih.gov/geo/query/acc.cgi?acc=GSE73129). To the best of our knowledge, this is the first report of microarray data for the paired and non-paired LBs, and the first report of the OE microarray data set analyzed in the present configuration of 16 SZ and 15 control subjects. The OE data have been reported previously in different configurations.^13, 28^

We examined expression patterns of co-regulated genes in the OE microarray data based on publicly available gene co-regulation networks.^29^ Raw data for the networks were downloaded from the Gene Expression Omnibus (accession id: GSE12526) and were processed to calculate the all-to-all correlation matrix as per the paper.^29^ A correlation coefficient cutoff of 0.75 re-constructed the networks consisting of 1645 correlations (that is, co-regulations) among 206 genes within a large set of human cell lines (*N*=295). Likewise, co-regulated gene pairs of the OE microarray data from only control subjects were also defined.

We compared the differentially expressed transcripts from the OE microarray to the genes identified by the Psychiatric Genomics Consortium as being associated with the 108 SZ-associated genetic loci.^30^ The 348 unique genes listed in the paper's 'Supplementary Table 3: Bioinformatic summary data for 108 genome-wide significant loci' were used to identify the 727 corresponding Affymetrix Human Genome U133 Plus 2.0 probe set IDs using Biomart (www.ensembl.org/biomart). A hypergeometric distribution was used to calculate whether the number of overlapping probe sets was beyond chance levels.

### Correlation analysis of neuropsychology test scores and OE molecular expression

In order to identify genes that may be associated with deficits in cognitive function in SZ, we filtered the differentially expressed genes in OE based on their correlation with neuropsychology test scores (the six-factor scores and composite score) from the SZ patients. The analysis was limited to SZ patients as we were interested in genes that specifically correlate with cognitive function in the disease state rather than cognition in general. The scores from SZ patients were normalized as *Z*-scores with adjustment for sex and years of education. Briefly, each raw score was regressed against sex and years of education in a linear model, yielding the expected score taking sex and years of education into consideration. The residual value was calculated using the expected score (residual value=actual score−expected score). The mean and the standard deviation (s.d.) of the residual values were used for calculating *Z*-scores [*Z*=(residual value−mean residual value)/s.d.]. Spearman's rank correlation analysis was performed to examine the relationship between differentially expressed genes and neuropsychology test *Z*-scores. *P*-values for the correlation tests were computed by permutation testing (*N*=1 000 000). R (http://www.r-project.org/, version 3.1.1) was used for correlation analysis.

### Real-time quantitative PCR data analysis

To validate the microarray data, gene expression was quantified using real-time quantitative polymerase chain reaction (qPCR) with a TaqMan Gene Expression Assay and ABI PRISM 7900HT Sequence Detection System (Applied Biosystems, Foster City, CA, USA) as per the manufacturer's instructions. Primers and probes were purchased from Life Technology (Carlsbad, CA, USA) [assay ID: *SMAD1*, Hs00195432_m1; *SMAD3*, Hs00706299_s1; *SMAD5*, Hs00289739_s1; SMAD specific E3 ubiquitin protein ligase 1 (*SMURF1*), Hs00410929_m1]. Human glyceraldehyde-3-phosphate dehydrogenase (*GAPDH*) was used as an internal control, and measurement of the threshold cycle (Ct) was performed in triplicate. Data were collected and analyzed with the Sequence Detector Software version 2.2 (Applied Biosystems) and the standard curve method. Relative gene expression was calculated as the ratio of the genes to the internal control (*GAPDH*). Outliers were determined as any data point more than 1.5 interquartile ranges below the first quartile or above the third quartile.

### Linear regression analysis

For evaluating the effects of antipsychotic medication on gene expression profiles, we converted daily doses of antipsychotics at the time of the biopsy to chlorpromazine equivalents.^31^ Then, we conducted linear regression analysis for gene expression levels detected by qPCR using the chlorpromazine equivalents as a covariate. Linear regression was also used to evaluate the effect of smoking (using the number of packs of cigarettes smoked per day as an independent variable) on gene expression levels in the qPCR experiments, and on the neuropsychological assessment scores.

## Results

**Figure 1:**
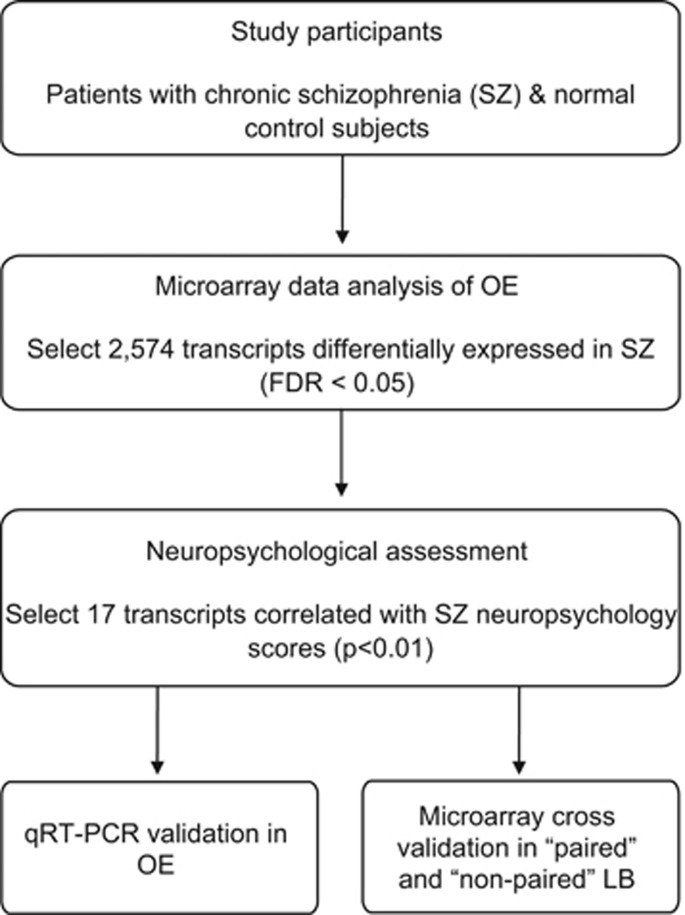
Study design. Microarray analysis using olfactory epithelium (OE) tissue and neuropsychological assessments were conducted in the same participants. Following differential gene expression analysis (false discovery rate (FDR) <0.05), correlation of gene expression with neuropsychological test scores was used as a filter to select candidate genes for validation with quantitative real-time PCR (qPCR). To determine whether genes identified using OE would also be significantly different in lymphoblasts (LBs), we conducted microarray differential gene expression analysis in 'paired' and 'non-paired' samples.

To investigate which genes or gene groups in neuronal cells are related to cognitive function in SZ, we designed the present study as follows (scheme shown in Figure 1).

### The demographics of the participants and clinical assessment

Sixteen patients with chronic SZ and fifteen normal control subjects participated in the neuropsychological testing and OE biopsy. Their demographic data and neuropsychological scores are shown in Table 1. The participants were matched for age (SZ, 39.8±11.3 years; control, 41.8±10.8 years; *P*=0.60), sex (SZ, 12 males and 4 females; control, 11 males and 4 females; *P*=0.77) and race (SZ, 6 Caucasian and 10 African-American; control, 6 Caucasian and 9 African-American; *P*=0.92). Years of education (SZ, 12.0±2.6; control, 13.4±2.6; *P*=0.16) and current smoking status (SZ, 9 yes and 7 no; control, 3 yes and 12 no; *P*=0.07) did not differ significantly. The cognitive assessments consist of six domains and the composite score. Among the six domains, there was a significant difference in performance across neurocognitive tests, with patients' performance being worse than healthy controls for processing speed, attention/working memory and ideational fluency (*P*=0.02, 0.04 and 0.004, respectively). The composite score was also significantly lower in patients (*P*=0.03). These differences remained significant even after adjusting for smoking status by linear regression analysis (data not shown). Patients underwent the Scales for the Assessment of Negative and Positive Symptom tests, with the mean scores being 6.6±4.1 and 3.8±3.6, respectively.

### Gene expression changes in SZ from LCM-enriched neural layers of OE

Following LCM, relative expression levels of the neuronal marker olfactory marker protein (OMP) were analyzed in a subset of control samples to confirm consistency of enrichment with previous studies (Supplementary Figure 1). Microarray analysis was performed on microdissected tissue from the same subjects who underwent the neuropsychological assessments. We identified 2574 transcripts that were differently expressed between SZ and control in olfactory neuronal tissues enriched by LCM FDR <0.05. The differentially expressed gene list, ordered by fold change, is available as Supplementary Table 1.

As validation of the OE microarray data, we examined the co-regulated genes in the OE tissues by comparing with those in the publicly available co-regulation networks consisting of 1645 gene–gene correlations (that is, co-regulations; |Pearson's coefficient|⩾0.75) within a large set of human cell lines (immortalized B cells from 295 normal individuals).^29^ The co-regulated genes of the OE tissues, which were defined as above, were significantly over-represented in the co-regulation networks (82/1645=4.98%, *P*=2.92 × 10^−41^; calculated by using hypergeometric distribution), suggesting that co-regulation patterns of the OE microarray data are shared, at least in part, with those within the human cell lines. In addition, we examined the relationship of the differentially expressed genes in the OE tissues with the co-regulation networks and found that few of the differential gene pairs exist on the co-regulation networks (3/1645<1% *P*≈1.00).

Forty-eight genes were overlapped between the probe sets associated with the 108 SZ-associated genetic loci^30^ and our 2574 differentially expressed OE transcripts (48/727=6.6%, *P*=0.0148; calculated by using hypergeometric distribution; Supplementary Table 1). None of the 17 genes correlated with neuropsychological test scores (described below) were represented in the overlap.

### Correlation between neuropsychological domain scores and gene expression

In order to narrow down from the 2574 differentially expressed genes (FDR<0.05) to those that may be specifically associated with cognitive function in SZ patients, we calculated Spearman's rank correlations between genes and patient neuropsychology test scores (composite and factor scores for individual domains). For this filtering method we used a cutoff of *P*<0.01, which narrowed our list of candidate genes to 17 (Table 2). As scores for the composite, processing speed, attention/working memory and ideational fluency domains were significantly lower in SZ patients compared with control, we focused on the genes that correlated with these four domains (*SMAD5, PABPC4L, RBM28, RAB4A, ERO1LB, CCL11, CD69, LINC01004, COA3, CBFA2T2*). Two genes, SMAD family member 5 (*SMAD5*) and poly(A) binding protein, cytoplasmic 4-like (*PABPC4L*), positively correlated with the composite score, which is the most representative of the neuropsychological assessment scores. *SMAD5* was also positively correlated with the processing speed score. Although its function is not well understood, a rare copy number variation in the *PABPC4L* gene was found in a treatment-resistant depression cohort in a recent study.^32^

### qPCR validation in OE and cross-validation in LB microarray

**Figure 2:**
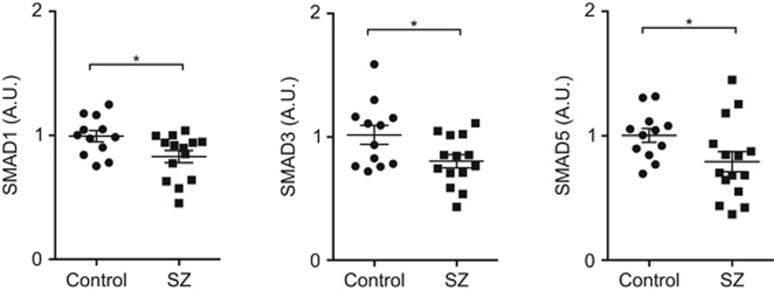
Gene expression level of SMAD pathway-related genes in the olfactory epithelium (OE) neuronal layer. The mRNA expression level of SMAD pathway-related genes using quantitative real-time polymerase chain reaction (qPCR). The qPCR analysis confirmed downregulation of SMAD1, SMAD3 and SMAD5 in schizophrenia (SZ). Expression levels were normalized to glyceraldehyde-3-phosphate dehydrogenase (GAPDH) expression. Results are shown as mean±s.e.m. Statistical analysis was conducted with Welch's *t*-test. *P<0.05.

As the *SMAD5* gene is located at chromosome 5q31.1, a suggested risk locus for SZ,^33, 34^ and has a role in neurodevelopment,^35^ we further explored expression of molecules in the SMAD pathway. The OE microarray demonstrated a downregulation of representative molecules from the SMAD pathway, such as *SMAD1*, *SMAD5* (both FDR<0.05), *SMAD3* and *SMURF1*, in SZ patients compared with controls. qPCR confirmed that *SMAD1*, *SMAD3* and *SMAD5* were differentially expressed in OE (*P*<0.05; Figure 2).

Exposure to tobacco smoke has been shown to change the binding activity of transcription factors, including SMAD3/4, in human lung cells.^36^ As olfactory epithelia, like lung tissue, would be directly exposed to cigarette smoke, the SMAD pathway in olfactory epithelia might be affected by cigarette smoke. To evaluate the relationship between SMAD gene expression and smoking, we conducted linear regression analysis involving SMAD gene expression (qPCR) as a dependent variable and the number of packs of cigarettes smoked per day as an independent variable. None of the SMAD genes showed a significant relationship with the number of packs smoked (*P*>0.05; data not shown).

We next asked whether OE neuronal tissues might have unique advantages as brain surrogates. Thus, we examined gene expression in blood cells from the same subjects to determine whether the same molecular signature (for example, downregulation of *SMAD5*) was also observed in peripheral cells. As described in the Materials and methods section, we used both 'paired' and 'non-paired' LBs. Strikingly, none of the 17 genes identified in OE tissue using the combined strategy of FDR cutoff and correlation with neuropsychology test scores were differentially expressed at FDR<0.05 between patients and controls in either the 'paired' or 'non-paired' LB data sets (Table 3). This result suggests that there is little overlap in the differential gene expression characteristics of OE and LB cells, supporting the utility of OE samples over peripheral cell samples in the analysis of brain-related changes.

## Discussion

This study aimed to develop a unique strategy for identifying neuronal changes associated with cognitive impairment in SZ at the molecular level. Cognitive deficits in SZ are intrinsic to the disorder, rather than being medication-induced. A meta-analysis of cognitive performance in antipsychotic-naive SZ patients has revealed impairments in processing speed and working memory.^37^ Furthermore, our research design, obtaining neuronal information from living patients, allows us to longitudinally track changes in gene expression that may be associated with changes in cognitive function, which is not possible using post-mortem brain tissue. We propose that the SMAD pathway may be an important target in studying cognitive deficits in SZ.

SMAD proteins are intracellular mediators in the bone morphogenic protein and transforming growth factor-beta (TGF-β) signaling pathways and are important in regulating neurodevelopment; SMAD2 and 3 are downstream of TGF-β receptors, whereas SMAD1, 5 and 8 are primarily substrates for the bone morphogenic protein receptors.^38, 39^ Dysregulation of *SMAD* genes reportedly affect oligodendrogenesis,^40^ axon development and regeneration,^40^ growth and maintenance of midbrain dopaminergic neurons,^41^ as well as differentiation, maintenance and protection of basal forebrain cholinergic neurons.^42, 43, 44, 45, 46, 47^ Cholinergic neurons are thought to influence diverse cognitive tasks including attention, learning and memory. There is growing evidence for the dysfunction of cholinergic systems in SZ and related disorders.^48, 49^ The SMADs are also critical for bone formation and intriguingly, bone function may be important for stability of brain function; in addition to being a structural organ, the bone functions as an endocrine organ by secreting osteocalcin, which has been suggested to have a direct role in memory and mood.^50, 51^ There have been several reports that include an alteration in the molecular cascade in various mental conditions,^34, 52, 53, 54, 55^ and the present study defines its implication in the cognition associated with SZ.

There is precedence for applying a multifaceted approach to explore biomarkers for mental illnesses. For example, the convergent functional genomics approach aims to increase the sensitivity and specificity of biomarkers by integrating data from animal models, genetics, gene expression studies in post-mortem brain and blood samples.^56, 57, 58, 59, 60, 61^ We feel that our approach of combining expression data from different human cell types, and filtering them based on correlation with a behavioral trait of interest, is complementary to the convergent functional genomics approach, and may be particularly informative in a translational psychiatry setting.

This exploratory study has some limitations. Although the two groups (SZ and control) are matched by age, race, sex, years of education and smoking status, we cannot exclude differences due to other confounding factors, such as the effects of long-term medication and diet. Note, as far as daily dose of antipsychotic medication at the time of biopsy is used as a covariate, linear regression analysis indicates that antipsychotic medication had no significant effect on gene expression levels (Supplementary Table 2). However, future studies with medication-naive patients would be informative. Although the current data suggest the SMAD pathway as a novel target in studying cognitive deficits in SZ, we fully acknowledge that the sample size of the present study is relatively small. Further studies with larger sample sizes are warranted.

Our unique approach of combining the analysis of two observations from the same subjects, microarray from LCM-processed OE neuronal tissues and neuropsychological assessment, allowed us to identify potentially important new gene associations. Of note, our results showed that the neuronal OE tissues provide unique molecular information that could not be obtained from blood cells (immortalized LBs) in the present data set, including the molecules of the SMAD pathway (Table 3). This failure is partly because the process for immortalization leads to the loss of state information in the fresh blood. It is also possible that blood studies may need larger sample size. However, this does not diminish the significance and utility of blood cells in molecular profiling in mental disorders: indeed, there is successful precedence in using blood cells for the profiling.^53, 54, 55, 56, 57, 58, 59, 60, 61^ At least within the present study, we can say that the neuronal OE tissues may be good for academic purposes and understanding the biology of illness, whereas the blood, in particular fresh blood, is advantageous for repeated routine monitoring and clinical practice. Thus, although negative in LBs, it may be an important future effort to study the SMAD pathway in the fresh whole blood together with neurocognition.

The present experimental strategy with OE neuronal tissues may be useful in translational psychiatry, particularly in studies that look for molecular signatures that reflect the 'state' at the time of biopsy. Although recent advances in stem cell biology, such as induced pluripotent stem cells, have given us the ability to generate neurons and glia from patients, these cells can capture only 'trait' changes.^12, 62, 63^ An ongoing challenge in translational psychiatry is to capture treatment-associated 'state' alterations in neuronal molecular markers that correlate with specific features in mental illness. For example, it is very important to understand molecular mechanisms underlying treatment response, or resistance to lithium, or neuroleptics. Although such application is beyond the scope of this study, our experimental strategy can be used to address this question—conducting biopsies before and after the treatment and comparing molecular and clinical changes in response to the treatment at the level of the individual.

Additionally with the maturation of next-generation sequencing technologies, it is becoming easier to obtain reliable transcriptomic data from small amounts of starting material. Beyond studies for treatment response to current medications, we anticipate that this experimental system can also be effectively utilized to identify new drug targets by connecting molecular/biological effects and clinical outcomes. The utility of this system will be further improved by the development of a platform for protein assays in future studies.

## Figures and Tables

**Table 1:** Participant demographic data and neuropsychology test data

*Characteristics*	*Main data set (paired OE and LB*^a^)			*Non-paired LB data set*		
	*SZ (*N=*16)*	*Control (*N=*15)*	P*-value*	*SZ (*N=*16)*	*Control (*N=*18)*	P*-value*
Age (years, average±s.d.)	39.8±11.3	41.8±10.8	0.60^b^	36.4±11.2	35.6±11.1	0.84^b^
Sex (male/female)	12/4	11/4	0.77^c^	11/5	14/4	0.70^c^
Race (Caucasian/African American/others)	6/10/0	6/9/0	0.92^c^	3/12/1	9/8/1	0.09^c^
Years of education (years, average±s.d.)	12.0±2.6	13.4±2.6	0.16^b^	12.3±2.4	12.9±1.9	0.35^b^
Smoking (yes/no/NA)	9/7/0	3/12/0	0.07^c^	10/5/1	5/13/0	0.04^c^
SANS (average±s.d.)	6.6±4.1			6.1±3.2		
SAPS (average±s.d.)	3.8±3.6			4.5±3.3		
						
*Neurocognitive function (average±s.d.)*						
Composite	8.6±1.5	9.9±1.6	0.03^b^			
Processing speed	8.9±2.0	11.0±2.4	0.02^b^			
Attention/working memory	8.7±2.1	10.6±2.6	0.04^b^			
Verbal learning and memory	9.6±1.9	9.8±2.6	0.79^b^			
Visual learning and memory	9.5±1.9	9.6±2.8	0.83^b^			
Executive functioning	9.3±2.4	10.7±2.5	0.14^b^			
Ideational fluency	5.7±1.8	7.8±1.7	0.004^b^			

Abbreviations: LB, lymphoblast; OE, olfactory epithelium; SANS, Scales for the Assessment of Negative Symptom; SAPS, Scales for the Assessment of Positive Symptom; SZ, schizophrenia.

a LB samples consist of 15 patients with SZ and 15 controls.

b *t*-test.

c Fisher's exact test.

**Table 2:** Correlation between neuropsychological domain scores in schizophrenia patients and differential gene expression in OE

*Neuropsychology domain*	*Gene symbol*	*Probe set ID*	*Gene name*	*Fold change in schizophrenia OE, compared to control OE*	*Spearman rho*	*Permutation test,* P*-value*
**Composite**	*SMAD5*	*225223_at*	SMAD family member 5	−1.2	0.65	0.004
	*PABPC4L*	*238865_at*	poly(A) binding protein, cytoplasmic 4-like	−2.2	0.63	0.005
**Processing speed**	*SMAD5*	*225223_at*	SMAD family member 5	−1.2	0.70	0.002
**Attention/working memory**	*RBM28*	*218593_at*	RNA binding motif protein 28	−1.4	0.72	0.001
	*RAB4A*	*203581_at*	RAB4A, member RAS oncogene family	1.3	−0.71	0.001
	*ERO1LB*	*231944_at*	ERO1-like beta (*S. cerevisiae*)	−1.3	0.69	0.002
	*CCL11*	*210133_at*	chemokine (C-C motif) ligand 11	−3.3	0.64	0.005
	*CD69*	*209795_at*	CD69 molecule	−2.5	0.64	0.005
Verbal learning and memory	*NGDN*	*216263_s_at*	neuroguidin, EIF4E binding protein	−2.0	0.64	0.004
	*SMAD5*	*225223_at*	SMAD family member 5	−1.2	0.62	0.006
Visual learning and memory	*PABPC4L*	*238865_at*	poly(A) binding protein, cytoplasmic 4-like	−2.2	0.78	0.000
	*SRGN*	*201859_at*	serglycin	−1.8	0.66	0.003
Executive functioning	*ZFP36L1*	*211962_s_at*	zinc finger protein 36, C3H type-like 1	−1.2	0.69	0.002
	*AP5S1*	*219706_at*	adaptor-related protein complex 5, sigma 1 subunit	1.4	−0.64	0.005
	*SERPINA12*	*1552544_at*	serpin peptidase inhibitor, clade A (alpha-1 antiproteinase, antitrypsin), member 12	1.02	−0.63	0.005
	*LYPLA2*	*215568_x_at*	lysophospholipase II	1.2	−0.63	0.005
	*SMC6*	*218781_at*	structural maintenance of chromosomes 6	−1.4	0.63	0.005
**Ideational fluency**	*LINC01004*	*235217_at*	long intergenic non-protein coding RNA 1004	−1.3	0.69	0.002
	*COA3*	*218026_at*	cytochrome c oxidase assembly factor 3	1.4	0.64	0.004
	*CBFA2T2*	*238549_at*	core-binding factor, runt domain, alpha subunit 2; translocated to, 2	−1.9	−0.64	0.004

Abbreviation: OE, olfactory epithelium.

**Table 3:** Expression changes of 17 genes from Table 2 in paired and non-paired lymphoblasts

*Gene symbol*	*Gene name*	*Probe set ID*	*Paired LB data set*		*Non-paired LB data set*	
			P*-value*	*Q-value*	P*-value*	*Q-value*
*SMAD5*	SMAD family member 5	225223_at	0.613	0.840	0.006	0.293
*RBM28*	RNA binding motif protein 28	218593_at	0.066	0.707	0.876	0.938
*RAB4A*	RAB4A, member RAS oncogene family	203581_at	0.897	0.965	0.426	0.663
*LINC01004*	long intergenic non-protein coding RNA 1004	235217_at	0.323	0.707	0.097	0.623
*ERO1LB*	ERO1-like beta (S. cerevisiae)	231944_at	0.698	0.882	0.793	0.894
*CCL11*	chemokine (C-C motif) ligand 11	210133_at	0.434	0.744	0.362	0.626
*NGDN*	neuroguidin, EIF4E binding protein	216263_s_at	0.780	0.919	0.432	0.667
*SRGN*	serglycin	201859_at	0.799	0.928	0.370	0.629
*ZFP36L1*	zinc finger protein 36, C3H type-like 1	211962_s_at	0.576	0.822	0.002	0.222
*AP5S1*	adaptor-related protein complex 5, sigma 1 subunit	219706_at	0.973	0.991	0.536	0.736
*COA3*	cytochrome c oxidase assembly factor 3	218026_at	0.970	0.990	0.227	0.623
*CBFA2T2*	core-binding factor, runt domain, alpha subunit 2; translocated to, 2	238549_at	0.196	0.707	0.062	0.623
*PABPC4L*	poly(A) binding protein, cytoplasmic 4-like	238865_at	0.817	0.935	0.611	0.785
*CD69*	CD69 molecule	209795_at	0.839	0.943	0.293	0.623
*SERPINA12*	serpin peptidase inhibitor, clade A (alpha-1 antiproteinase, antitrypsin), member 12	1552544_at	0.953	0.984	0.586	0.769
*LYPLA2*	lysophospholipase II	215568_x_at	0.408	0.731	0.623	0.793
*SMC6*	structural maintenance of chromosomes 6	218781_at	0.303	0.707	0.369	0.629

Abbreviation: LB, lymphoblast.
