# Supplementary material for: Molecular signatures associated with cognitive deficits in schizophrenia: a study of biopsied olfactory neural epithelium
Source: Transl Psychiatry. 2016 Oct 11;6(10):e915–. doi: 10.1038/tp.2016.154 (PMC5315541; doi:10.1038/tp.2016.154)
Supplement: Supplementary Figure 1 [file tp2016154x1.pdf]

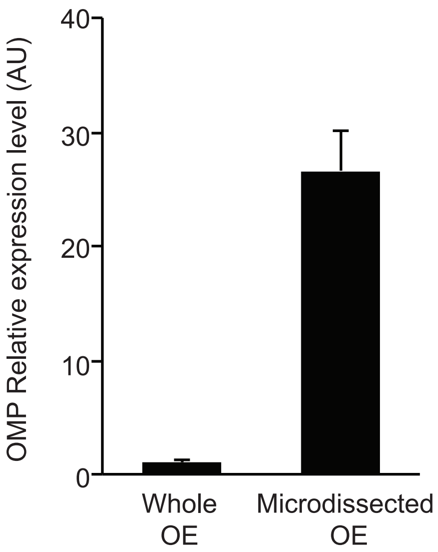


**Supplementary Figure 1: Enrichment of neuronal cells from nasal biopsy samples by laser-captured microdissection**

Quantitative real-time PCR for olfactory marker protein (OMP, a marker for olfactory receptor neurons). After microdissection, the relative expression level of OMP was nearly 30-fold higher in the microdissected tissue compared with that in the whole olfactory epithelium (OE) in test samples.
