# Supplementary material for: Molecular signatures associated with cognitive deficits in schizophrenia: a study of biopsied olfactory neural epithelium
Source: Transl Psychiatry. 2016 Oct 11;6(10):e915–. doi: 10.1038/tp.2016.154 (PMC5315541; doi:10.1038/tp.2016.154)
Supplement: Supplementary Table 3 [file tp2016154x4.pdf]

Supplementary Table 3 Linear regression model for neuroleptic effect on gene expression level

Gene symbol	Beta	95% CI (lower)	95% CI (upper)	*p*-value
SMAD1	-1.66E-04	-6.68E-04	3.36E-04	0.47
SMAD3	-8.50E-05	-5.53E-04	3.83E-04	0.69
SMAD5	1.26E-04	-5.37E-04	7.89E-04	0.68
SMURF1	-3.57E-04	-8.38E-04	1.25E-04	0.13
